# Obstructive Jaundice due to a Periportal Tuberculous Mass Complicated by Anti‐Tuberculous Drug‐Induced Liver Injury: A Case Report From a Low‐Resource Setting

**DOI:** 10.1002/ccr3.73443

**Published:** 2026-09-06

**Authors:** Girma Deshimo Lema, Yemisrach Chanie, Mathewos Tesfaye Yanore, Segenet Bizuneh Mengistu, Amanuel Berhanu Kotisso, Dawit Zena, Saymen Tsegaye Gebreeyesus, Tadesse Mulusew Gared, Nebyu Samuel Chekol, Ebrahim Umer Muhammed

**Affiliations:** ^1^ Department of Internal Medicine Debre Berhan University Debre Berhan Ethiopia; ^2^ Department of Internal Medicine St. Paul's Hospital Millennium Medical College Addis Ababa Ethiopia; ^3^ Department of Internal Medicine University of Gondar Gondar Ethiopia; ^4^ Department of Internal Medicine Arsi University Asella Ethiopia; ^5^ Department of Internal Medicine Aksum University Aksum Ethiopia; ^6^ Department of Radiology St. Paul's Hospital Millennium Medical College Addis Ababa Ethiopia; ^7^ Department of Internal Medicine Adama Hospital Medical College Adama Ethiopia

**Keywords:** ATDILI, obstructive jaundice, periportal tuberculous lymphadenitis, resource‐limited setting

## Abstract

Periportal tuberculous lymphadenitis with obstructive jaundice is rare and may mimic malignancy. We report a case of obstructive jaundice caused by a periportal tuberculous mass confirmed by fine‐needle aspiration and GeneXpert. Treatment was complicated by anti‐tuberculous drug‐induced liver injury. Tuberculosis should be considered in the differential diagnosis in endemic settings.

## Key Clinical Message

Periportal tuberculous lymphadenitis is a rare cause of obstructive jaundice that can mimic pancreaticobiliary malignancy. In tuberculosis‐endemic, resource‐limited settings, it should be considered in patients with periportal masses causing biliary obstruction. Anti‐tuberculous drug‐induced liver injury may complicate management, requiring prompt recognition, treatment interruption, close monitoring, and sequential drug reintroduction.

**Abbreviations**
ALP: alkaline phosphatase
ALT: alanine aminotransferase
AST: aspartate aminotransferase
ATDILI: anti‐tuberculous drug‐induced liver injury
CBD: common bile duct
CT: computed tomography
RHZE: rifampicin isoniazid, pyrazinamide, and ethambutol
TB: tuberculosis

## 1 Introduction

Tuberculosis (TB) remains a major global public health challenge and a leading cause of death from a single infectious agent worldwide. Most TB cases occur in high‐burden countries, particularly in Sub‐Saharan Africa, and Ethiopia remains among the World Health Organization‐designated high TB burden countries despite ongoing control efforts [1, 2].

Although pulmonary tuberculosis is the most common form, extrapulmonary TB can involve almost any organ [3, 4]. However, periportal lymph node involvement causing obstructive jaundice is rare and typically results from external compression of the biliary tract by tuberculous masses or lymphadenopathy in the periportal region. Clinical and radiological findings can mimic pancreaticobiliary malignancy or other inflammatory hepatobiliary diseases, making diagnosis challenging and frequently requiring histopathological confirmation [5, 6, 7, 8, 9].

Anti‐tuberculous therapy is the mainstay of treatment for both pulmonary and extrapulmonary tuberculosis [10]. However, its use may be complicated by anti‐tuberculous drug‐induced liver injury (ATDILI), particularly in patients with underlying hepatobiliary disease or extrapulmonary tuberculosis [11, 12, 13, 14]. Hepatotoxicity may require temporary interruption of therapy and cautious sequential drug reintroduction, resulting in delayed treatment and added management challenges, especially in low‐resource settings with limited diagnostic and monitoring capacity. The coexistence of obstructive jaundice due to isolated periportal tuberculous mass and ATDILI is rarely reported and poses substantial diagnostic and therapeutic challenges [15].

This case report describes a patient with obstructive jaundice caused by a periportal tuberculous mass complicated by anti‐tuberculous drug‐induced liver injury, highlighting the diagnostic and therapeutic challenges associated with this rare presentation of extrapulmonary tuberculosis. By illustrating the overlap between hepatobiliary tuberculosis and pancreaticobiliary malignancies, as well as the complexities of managing treatment‐related hepatotoxicity, this case emphasizes the importance of maintaining a high index of suspicion for tuberculosis in endemic settings and the need for careful monitoring and sequential drug reintroduction in resource‐limited environments.

## 2 Case History/Examination

A 23‐year‐old male patient presented to Saint Paul's Hospital Millennium Medical College in Addis Ababa with periumbilical and epigastric abdominal pain of 2 months' duration. He also reported night sweats, loss of appetite, and unquantified weight loss. Two weeks before presentation, he developed progressive yellowish discoloration of the eyes and dark urine.

Otherwise, he had no fever, pruritus, cough, chest pain, or change in bowel habits, and no history of contact with chronic coughers. There was no history of previous tuberculosis treatment or known chronic illness. He also had no history of smoking, significant alcohol intake, or drug use. His family history was unremarkable.

On physical examination, the patient had deeply icteric sclerae. His vital signs were within normal limits. He was underweight, with a body mass index of 18 kg/m^2^. Abdominal examination revealed mild epigastric tenderness, whereas the remainder of the systemic examination was unremarkable.

## 3 Differential Diagnosis, Investigations, and Treatment

Initial laboratory investigations revealed a total bilirubin level of 13.8 mg/dL; direct bilirubin measurement was not available at the time of testing. Alkaline phosphatase (ALP) was 416 U/L, alanine aminotransferase (ALT) was 118 U/L, and aspartate aminotransferase (AST) was 63.4 U/L. Alpha‐fetoprotein (AFP) was mildly elevated at 11.7 ng/mL (reference range: 0–5.8 ng/mL). Complete blood count (CBC), erythrocyte sedimentation rate (ESR), random blood sugar, lipase, amylase, and other serum chemistry parameters were within normal limits. Serologic tests for human immunodeficiency virus (HIV), hepatitis B virus, and hepatitis C virus were negative.

Abdominal ultrasound revealed a 4.7 × 3.35 cm complex multiloculated mass at the head of the pancreas causing mass effect on the common bile duct (CBD) and portal vein (PV), with associated dilatation of the intrahepatic and extrahepatic biliary ducts. A triphasic computed tomography (CT) scan was subsequently performed for further characterization of the lesion and additional evaluation.

**FIGURE 1:**
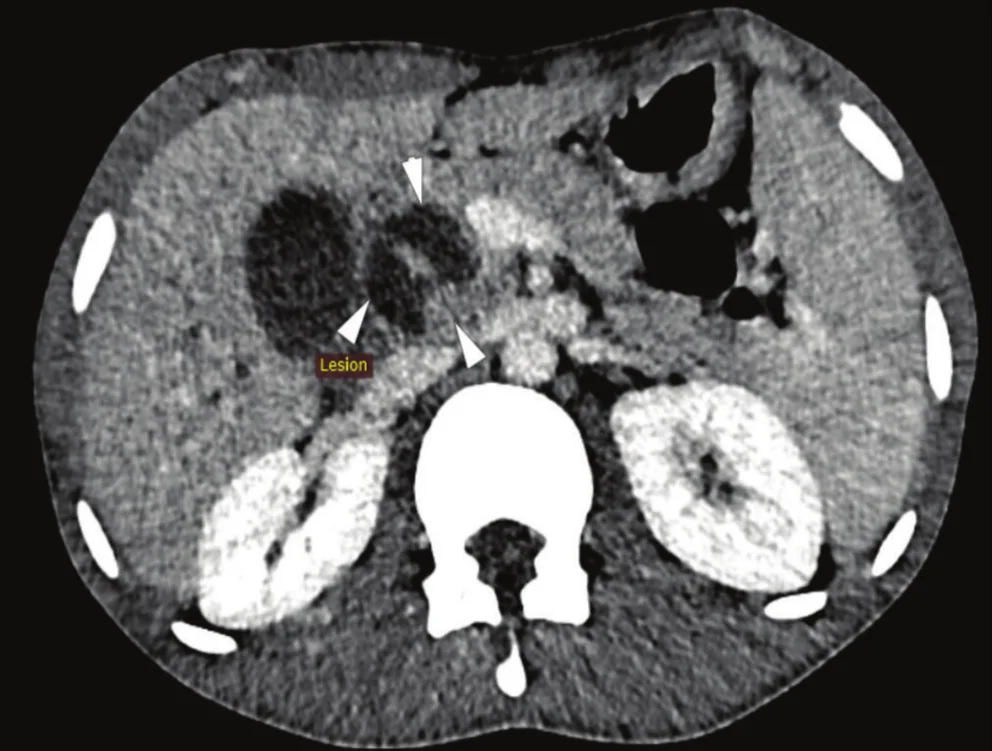
Axial contrast‐enhanced computed tomography image at the level of the porta hepatis showing a poorly circumscribed, heterogeneously attenuating periportal mass lesion measuring 3.3 × 3.5 cm, with heterogeneous contrast enhancement and a prominent non‐enhancing component.

**FIGURE 2:**
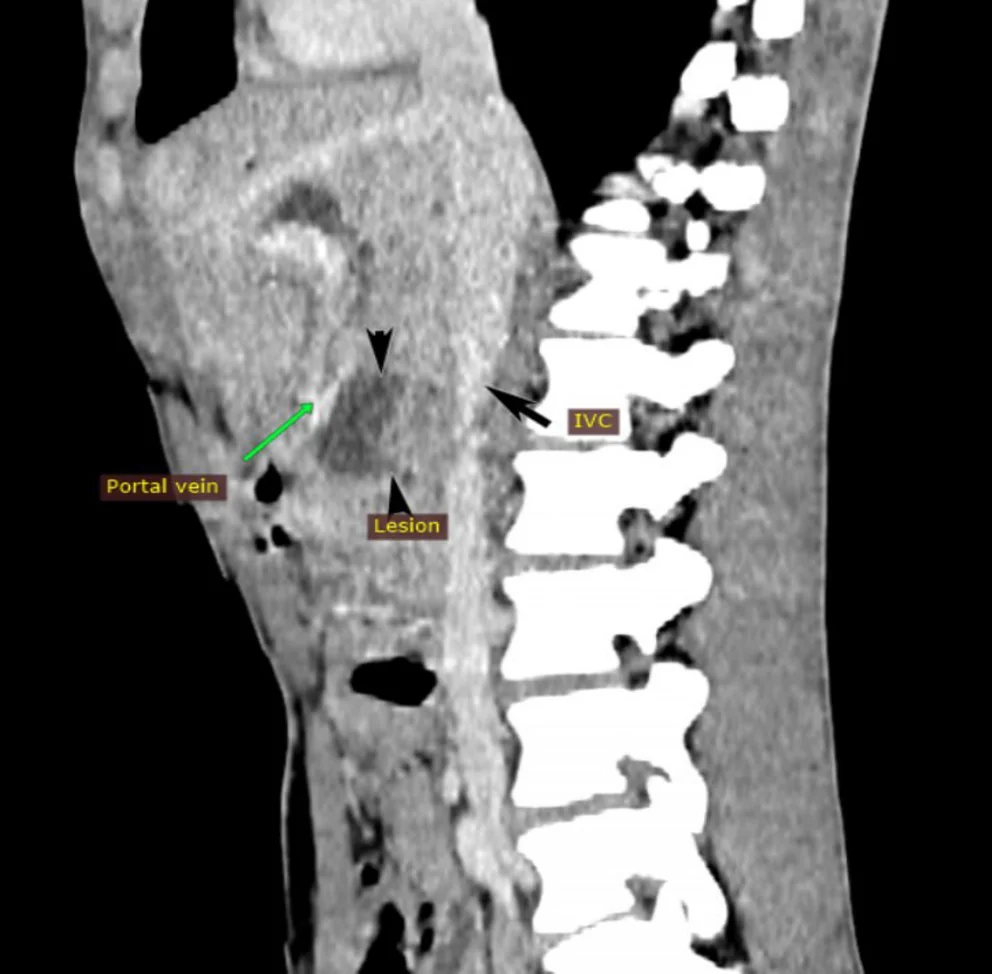
Sagittal contrast‐enhanced CT reconstruction through the porta hepatis demonstrating posterior compression of the inferior vena cava (IVC) and anterior displacement of the main portal vein by the periportal lesion.

**FIGURE 3:**
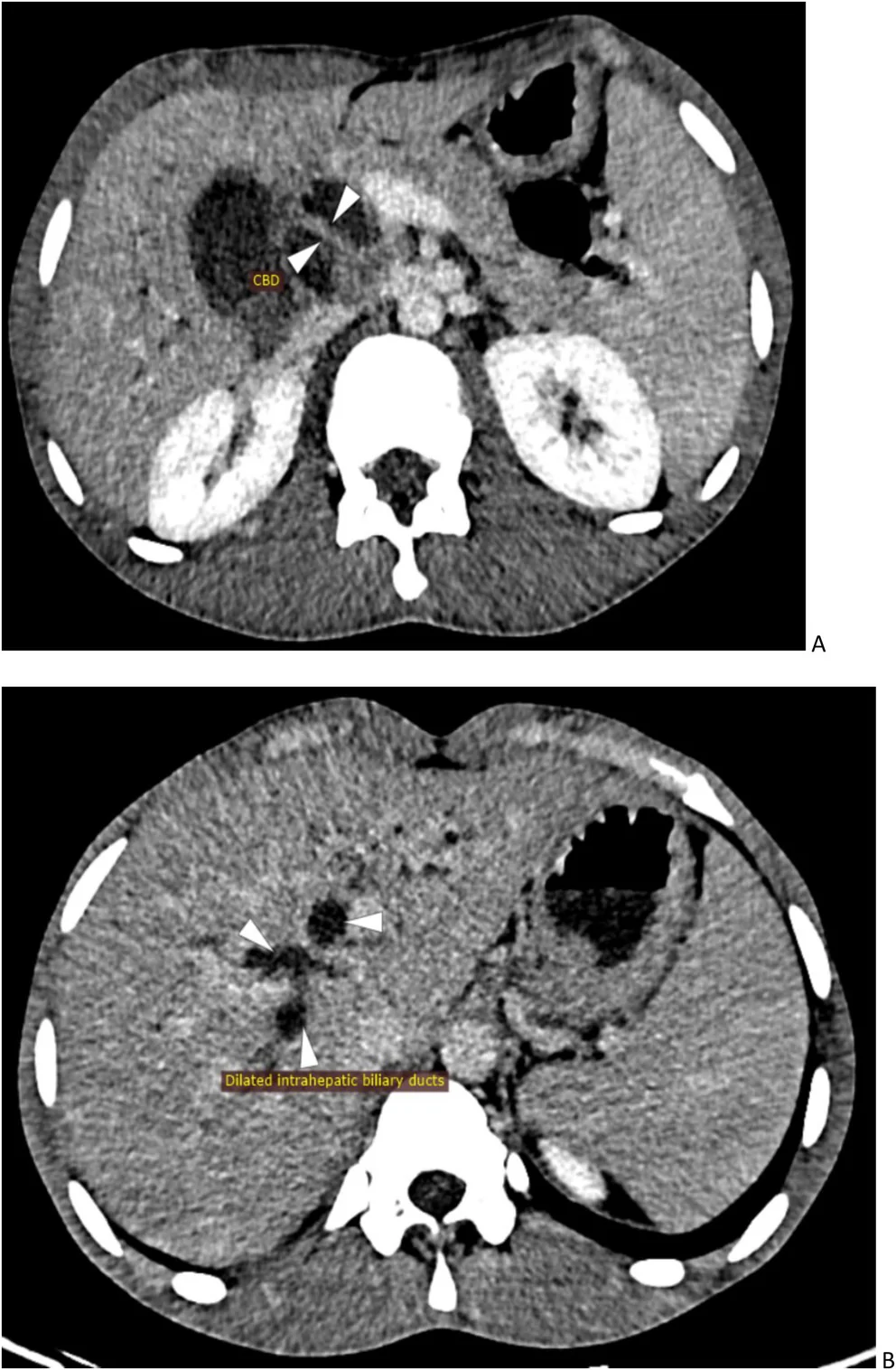
(A, B) Axial contrast‐enhanced CT image at the level of the common bile duct demonstrating narrowing of the mid common bile duct as it traverses the periportal lesion (A), resulting in upstream intrahepatic biliary duct dilatation (B).

The CT scan demonstrated a poorly circumscribed heterogeneously attenuating periportal mass lesion measuring 3.3 × 3.5 cm, showing contrast enhancement with a prominent non‐enhancing component (Figure 1). The epicenter of the lesion was located at the foramen of Winslow, causing posterior compression of the inferior vena cava (IVC) and anterior displacement of the main portal vein (Figure 2). The mid‐segment of the CBD was seen traversing the mass lesion with significant luminal narrowing, resulting in upstream biliary ductal dilatation (Figure 3A,B). His chest X‐ray was unremarkable.

Based on these imaging findings, confluent periportal lymphadenopathy secondary to tuberculosis, lymphoma, or metastatic disease was considered. The case was presented at a multidisciplinary team (MDT) discussion, and an image‐guided biopsy was recommended.

An ultrasound‐guided fine‐needle aspiration (FNA) was subsequently performed from the lesion. Cytology demonstrated granulomatous inflammation consistent with tuberculosis, although the cytology slide was later unavailable for review. GeneXpert MTB/RIF assay was positive for *Mycobacterium tuberculosis* and rifampicin resistance was not detected.

With a final diagnosis of obstructive jaundice secondary to periportal tuberculous lymphadenitis, standard four‐drug anti‐tuberculous therapy was initiated according to the patient's weight. The need for biliary drainage was considered; however, it was decided to start anti‐tuberculous therapy without drainage, with close monitoring of liver function.

On day six of anti‐tuberculous treatment, the patient developed right upper quadrant abdominal pain, nausea, and vomiting. Repeat laboratory investigations showed a total bilirubin level of 17.3 mg/dL, ALP of 305 U/L, ALT of 883 U/L, AST of 1085 U/L, and an international normalized ratio (INR) of 1.79. CBC and renal function tests remained within normal limits. Based on the temporal relationship between initiation of anti‐tuberculous therapy and the onset of nausea, vomiting, and marked elevation of serum aminotransferases, together with the exclusion of alternative causes, a diagnosis of hepatocellular ATDILI was made. Anti‐tuberculous medications were withheld, and liver function tests were subsequently monitored closely. Pertinent laboratory trends are summarized in Table 1.

**TABLE 1:** Summary of trends in key laboratory investigations.

Follow up dates		Total bilirubin	Direct bilirubin	ALP	ALT	AST	INR	Alb
Baseline	Pre‐ATT	13.8	—	416	118	63.4	—	—
On ATT	At Day 6 of ATT^a^	17.3	—	305	883	1085	1.79	—
ATT discontinuation phase	At Day 10	9.2	—	194	205	51.8	—	—
	At Week 2	4.6	3.5	205	—	51.3	0.96	—
ATT reintroduction phase	At Day 3	3.9	—	191	—	43.5	—	—
	At Day 7	2.8	—	180	—	42.5	—	—
	At Week 2	2.1	—	95	40	38	—	3.96
	At Week 4	1.2	—	95	22	27.3	—	—
	At Week 8	1.12	—	—	—	—	—	—
	At Week 12	1.06	—	98	36	40	1.13	4.63
Reference ranges		0.2–1.2 mg/dL	0–0.3 mg/dL	55–149 U/L	0–41 U/L	0–40 U/L	0–1.3	3.2–4.5 mg/dL

Abbreviations: Alb, albumin; ALP, alkaline phosphatase; ALT, alanine aminotransferase; AST, aspartate aminotransferase; ATT, anti‐tuberculous therapy; INR, international normalized ratio.

^a^ ATT was discontinued at Day 6 due to suspected ATDILI.

The patient was managed supportively with a proton pump inhibitor and maintenance intravenous fluids. Following discontinuation of anti‐tuberculous therapy, his nausea and vomiting subsided, and liver chemistries gradually improved. At Week 2 after withholding anti‐tuberculous therapy, laboratory tests showed significant improvement, with total bilirubin decreasing to 4.6 mg/dL, direct bilirubin to 3.5 mg/dL, ALP to 205 U/L, and AST to 51.3 U/L, which were lower than the pre‐treatment levels.

At this stage, anti‐tuberculous therapy was cautiously reintroduced with rifampicin and isoniazid (RH) at a dose of two tablets daily for 3 days, followed by four tablets daily for 5 days. No worsening of liver function was observed, and full‐dose four‐drug therapy consisting of rifampicin, isoniazid, pyrazinamide, and ethambutol (RHZE) was subsequently restarted. The patient tolerated the regimen well, with progressive improvement in liver enzymes, resolution of jaundice, improved appetite, and no further episodes of vomiting.

## 4 Outcome and Follow Up

**FIGURE 4:**
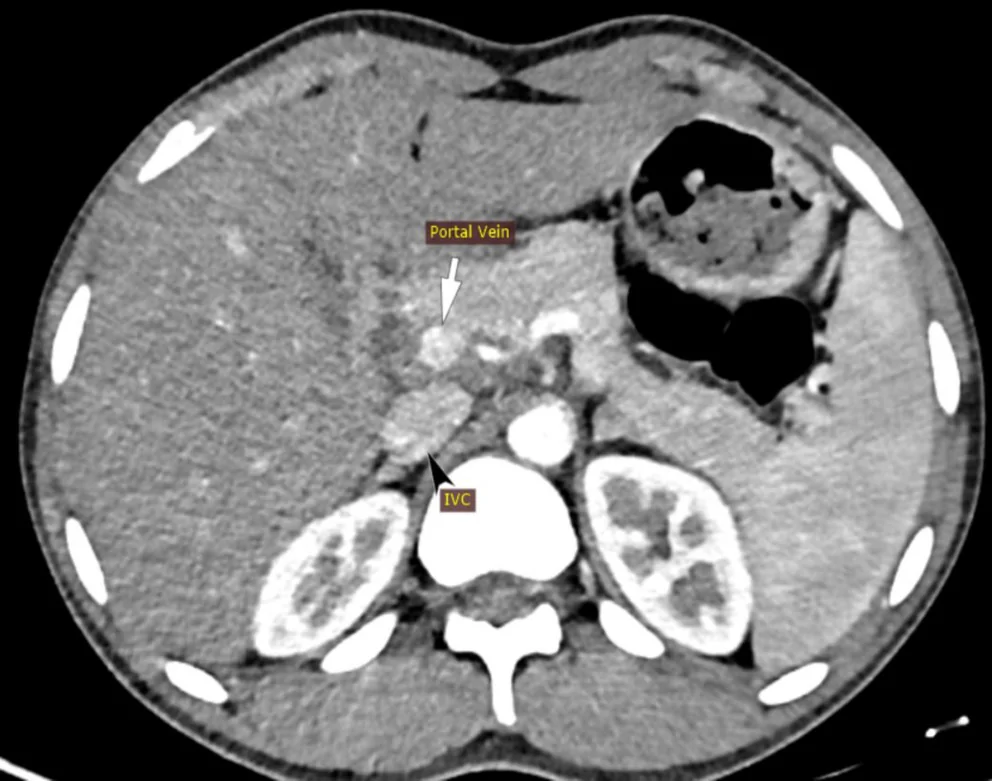
Axial contrast‐enhanced CT image at the level of the porta hepatis, obtained 10 weeks after treatment initiation, demonstrating complete resolution of the periportal lesion and marked improvement of the intrahepatic biliary dilatation.

Following sequential reintroduction of anti‐tuberculous therapy, liver function tests were monitored regularly throughout his 3‐week hospital stay and after discharge (Table 1). No recurrence of hepatotoxicity occurred. Liver enzymes and bilirubin progressively normalized, with complete normalization after 1 month of treatment. The patient's jaundice resolved completely, appetite improved, and he remained clinically stable throughout follow‐up. Follow‐up CT scan performed after 10 weeks of anti‐tuberculous therapy demonstrated complete resolution of the periportal lesion and marked improvement of biliary dilatation (Figure 4). At the latest follow‐up, he continued anti‐tuberculous therapy without further complications.

## 5 Discussion

Periportal tuberculous lymphadenitis presenting with obstructive jaundice is a rare manifestation of extrapulmonary tuberculosis. Although abdominal tuberculosis commonly affects the gastrointestinal tract, peritoneum, lymph nodes, solid abdominal organs, and biliary system [4, 5, 16, 17, 18], isolated periportal involvement leading to biliary obstruction is infrequently reported in clinical practice [15]. Obstructive jaundice is an uncommon complication of abdominal tuberculosis in general. In most reported cases, jaundice results from external compression of the common bile duct by enlarged periportal lymph nodes or tuberculous masses, as observed in our patient. Other proposed mechanisms of biliary obstruction in abdominal tuberculosis include tuberculous strictures of the biliary tree, pancreatic head tuberculosis, and retroperitoneal tuberculous masses [8, 9, 16, 19, 20, 21, 22].

Because the clinical manifestations and imaging findings often resemble pancreaticobiliary malignancies such as cholangiocarcinoma, pancreatic carcinoma, or lymphoma, establishing the diagnosis can be particularly challenging. Our patient initially presented with nonspecific constitutional symptoms, including night sweats, anorexia, and weight loss, in association with progressive jaundice. Such presentations are nonspecific and may mimic neoplastic or inflammatory hepatobiliary and pancreatic diseases as described in previous reports [8, 9, 16, 19, 20, 22]. There were no identifiable risk factors for tuberculosis in this patient apart from living in a TB‐endemic country.

An additional diagnostic challenge in our patient was the initially nonspecific imaging appearance of the lesion. The abdominal ultrasound evaluation was nonspecific, showing a complex multiloculated mass in the pancreatic head region causing biliary obstruction, without clearly defining the origin or nature of the lesion. Subsequent triphasic CT better characterized the lesion as a heterogeneously enhancing periportal mass with necrotic components, favoring confluent periportal lymphadenopathy over a primary pancreatic malignancy. Accordingly, the differential diagnosis included tuberculosis, lymphoma, and metastatic nodal disease. In the setting of constitutional symptoms and a TB‐endemic region, tuberculous lymphadenitis became a major consideration. Previous reports highlight abdominal tuberculosis as an important differential diagnosis of periportal masses and obstructive jaundice, especially in TB‐endemic regions [8, 9, 16]. Following MDT discussion, ultrasound‐guided FNA was performed, which demonstrated granulomatous inflammation, whereas GeneXpert MTB/RIF confirmed *Mycobacterium tuberculosis* . In low‐resource settings such as Ethiopia, image‐guided tissue sampling remains essential for establishing the diagnosis and avoiding unnecessary surgical intervention. GeneXpert MTB/RIF has been used in previous reports [8, 16, 23] and was crucial in confirming the diagnosis in this paucibacillary extrapulmonary setting, where conventional microbiology has limited yield. It also rapidly assessed rifampicin resistance, which was not detected in our case, supporting initiation of standard first‐line anti‐tuberculous therapy.

Management of tuberculous biliary obstruction is not well standardized because of the rarity of the condition. Some patients require biliary decompression through endoscopic, percutaneous, or surgical intervention, particularly in severe obstruction or cholangitis [7, 23, 24, 25], whereas others improve with anti‐tuberculous therapy alone [8, 9, 16, 26]. In our patient, despite marked jaundice and radiologic evidence of biliary obstruction, a conservative approach without biliary drainage was adopted following multidisciplinary discussion, as the patient remained hemodynamically stable, showed no clinical evidence of ascending cholangitis, and access to biliary drainage services was limited. The marked radiologic and biochemical improvement observed after initiation of anti‐tuberculous therapy further supported the effectiveness of this conservative approach. This case highlights that selected patients with tuberculous obstructive jaundice can be effectively managed with anti‐tuberculous therapy alone and careful clinical monitoring, particularly in resource‐limited settings.

The optimal anti‐tuberculous regimen in patients with tuberculous biliary obstruction is also not well standardized [8, 16]. In the present case, standard first‐line anti‐tuberculous therapy consisting of rifampicin, isoniazid, pyrazinamide, and ethambutol was initiated at full doses. Although the patient had markedly elevated bilirubin levels, baseline AST and ALT elevations were mild (< 3 times the upper limit of normal). In addition, access to alternative hepatoprotective agents such as moxifloxacin was limited, as these medications are primarily available in MDR‐TB treatment centers.

A major challenge in this case was the development of ATDILI shortly after initiation of therapy. ATDILI is one of the most significant adverse effects associated with first‐line anti‐tuberculous agents, particularly isoniazid, rifampicin, and pyrazinamide [14, 27, 28, 29, 30]. Diagnosis can be particularly challenging in patients with underlying hepatobiliary disease, as worsening jaundice may result from progression of biliary obstruction, drug‐induced hepatotoxicity, or a combination of both. In our patient, the diagnosis of ATDILI was supported by the close temporal relationship between initiation of anti‐tuberculous therapy and the development of gastrointestinal symptoms with a marked hepatocellular pattern of liver injury, exclusion of alternative causes, and rapid biochemical improvement after drug withdrawal. The coexistence of obstructive jaundice and hepatotoxicity posed a considerable diagnostic and therapeutic challenge.

Current recommendations for ATDILI management emphasize immediate discontinuation of hepatotoxic drugs followed by sequential or full dose reintroduction once liver enzymes improve [10, 12, 30, 31]. In our patient, anti‐tuberculous therapy was withheld and liver function was closely monitored until significant biochemical recovery occurred. Sequential reintroduction beginning with rifampicin and isoniazid was tolerated without recurrence of hepatotoxicity, allowing successful reinitiation of full‐dose RHZE therapy. This favorable outcome highlights the importance of careful clinical and biochemical monitoring during rechallenge, particularly where alternative anti‐tuberculous regimens and specialized hepatology support are limited.

Finally, clinicians should maintain a high index of suspicion for ATDILI in patients who clinically deteriorate after initiation of anti‐tuberculous therapy, especially in the presence of underlying hepatobiliary disease.

## 6 Conclusion

In conclusion, this report describes a rare case of obstructive jaundice caused by periportal tuberculous lymphadenitis complicated by anti‐tuberculous drug‐induced liver injury. The case highlights the diagnostic overlap between hepatobiliary tuberculosis and malignancy, the challenges of managing biliary obstruction without advanced interventional resources, and the complexity of distinguishing worsening obstruction from treatment‐related hepatotoxicity. Early recognition, tissue diagnosis, close biochemical monitoring, and cautious sequential drug reintroduction were critical to achieving a successful outcome.

## Author Contributions

**Girma Deshimo Lema:** conceptualization, data curation, formal analysis, methodology, investigation, writing – review and editing, writing – original draft, resources, validation, supervision. **Yemisrach Chanie:** data curation, formal analysis, investigation, validation, supervision. **Mathewos Tesfaye Yanore:** conceptualization, data curation, formal analysis, investigation, writing – original draft. **Segenet Bizuneh Mengistu:** formal analysis, data curation, investigation, validation, supervision. **Amanuel Berhanu Kotisso:** data curation, methodology, investigation, validation. **Dawit Zena:** investigation, methodology, validation, data curation. **Saymen Tsegaye Gebreeyesus:** investigation, methodology, validation, data curation. **Tadesse Mulusew Gared:** data curation, investigation, formal analysis, supervision, validation. **Nebyu Samuel Chekol:** data curation, methodology, investigation, validation. **Ebrahim Umer Muhammed:** conceptualization, data curation, formal analysis, investigation, writing – original draft.

## Funding

The authors have nothing to report.

## Consent

Written informed consent was obtained from the patient for publication of this case report and any accompanying images.

## Conflicts of Interest

The authors declare no conflicts of interest.

## Data Availability

The data that support the findings of this study are available from the corresponding author upon reasonable request.
